# Societal causes of, and responses to, ocean acidification

**DOI:** 10.1007/s13280-018-1103-2

**Published:** 2018-11-14

**Authors:** Sverker C. Jagers, Simon Matti, Anne-Sophie Crépin, David Langlet, Jonathan N. Havenhand, Max Troell, Helena L. Filipsson, Victor R. Galaz, Leif G. Anderson

**Affiliations:** 1 Department of Political Science, University of Gothenburg, Box 711, Sprängkullsgatan 19, 405 30 Göteborg, Sweden; 2 Political Science Unit, Luleå University of Technology, 97187 Luleå, Sweden; 3 Department of Geology, Lund University, 22362 Lund, Sweden; 4 Department of Marine Sciences-Tjärnö, Tjärnö Marine Laboratory, University of Gothenburg, 45296 Strömstad, Sweden; 5 Department of Law, University of Gothenburg, Box 650, 40530 Göteborg, Sweden; 6 The Beijer Institute of Ecological Economics, The Royal Swedish Academy of Science, Lilla Frescativägen 4, 104 05 Stockholm, Sweden; 7 Stockholm Resilience Centre, Stockholm University, Kräftriket 2 B, 10691 Stockholm, Sweden; 8 Department of Marine Sciences, University of Gothenburg, Box 461, 40530 Göteborg, Sweden

**Keywords:** Adaptation, Causes, Governance, Markets, Mitigation, Ocean acidification

## Abstract

Major climate and ecological changes affect the world’s oceans leading to a number of responses including increasing water temperatures, changing weather patterns, shrinking ice-sheets, temperature-driven shifts in marine species ranges, biodiversity loss and bleaching of coral reefs. In addition, ocean pH is falling, a process known as ocean acidification (OA). The root cause of OA lies in human policies and behaviours driving society’s dependence on fossil fuels, resulting in elevated CO_2_ concentrations in the atmosphere. In this review, we detail the state of knowledge of the causes of, and potential responses to, OA with particular focus on Swedish coastal seas. We also discuss present knowledge gaps and implementation needs.

## Introduction

Current emissions of anthropogenic carbon dioxide (CO_2_) generate increasing temperatures, changing weather patterns, shrinking ice-sheets, a poleward shift of marine species ranges (Doney et al. 2012; García Molinos et al. 2016), and an increase in the amount of CO_2_ dissolved in the oceans leading to lower ocean pH—a process known as ocean acidification (OA).^1^ Anthropogenic OA occurs when increasing concentrations of atmospheric CO_2_ dissolve in the surface water to form carbonic acid. OA is identified as a global environmental problem in the United Nations’ Sustainable Development Goal 14.3, and has been classified as one of the nine planetary boundaries of importance for regulating the stability of the Earth’s system (Rockström et al. 2009). It is a global issue as its cause is the rising amounts of CO_2_ in the almost perfectly mixed atmosphere, even though emission patterns differ locally. The effects of OA are geographically highly heterogeneous and uncertain (Steffen et al. 2015). Although the understanding of the natural science processes underpinning OA, and consequences thereof, is continuously improving (Doney et al. 2012; Cooley et al. 2015; Gaylord et al. 2015; Riebesell and Gattuso 2015; Osborne et al. 2017), there is a general lack of understanding and public debate on the societal causes and policy options in most countries in relation to OA. This lack of understanding critically affects the ability to project—and adapt to—the likely effects of these changes on important ecosystem services provided by the seas, e.g. fisheries, coastal protection, nutrient recycling, recreation, and tourism. Hence contributions to increasing OA, and the measures taken to reduce acidification or to alleviate its societal consequences, vary considerably between contexts.

**Fig. 1:**
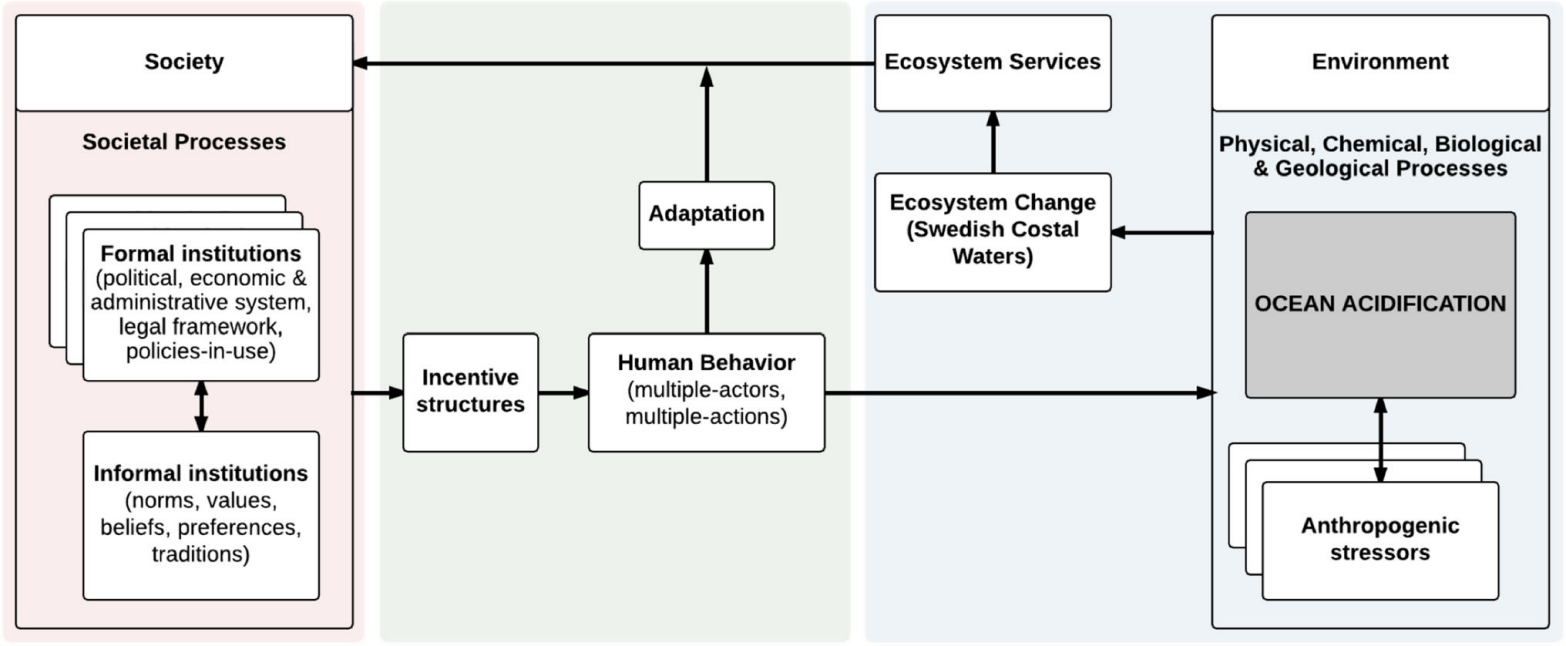
Schematic representation of ocean acidification, focus of this paper in grey. (Author names withheld) Focuses on the remaining parts

Figure 1 illustrates how OA interacts with the tight connections between society and the natural environment. Existing formal and informal institutions create incentive structures that frame and limit the preferences and actions of individuals. People’s actions affect the natural environment in various ways, for example, increasing levels of CO_2_ in the atmosphere. Further, the resulting OA risks affecting society by changing ecosystem structure and functions and hence influencing the services that these ecosystems produce.

This interdisciplinary review therefore targets the societal aspects of OA, primarily, legal, political, and economic aspects. It forms a complementary contribution to the growing literature which focuses on the environmental impacts of OA, and to which we refer for more details on these aspects (e.g. Doney et al. 2009; Hönisch et al. 2012; Riebesell and Gattuso 2015; Osborne et al. 2017). We summarize current knowledge about major anthropogenic causes of OA, and what the social sciences have pointed out as needed primary responses to mitigate these causes and alleviate OA’s direct and indirect consequences. Three questions have guided our joint efforts:

(Q1) What are the primary causes of anthropogenic ocean acidification from a social science perspective?

(Q2) How can society and politics respond to ocean acidification?

(Q3) What are the major knowledge gaps and research needs in the social sciences with regard to the future study of OA?

Despite its potentially severe negative implications for nature and society, the acidification of our coastal and ocean waters is seldom highlighted in media, public debate, or within national environmental politics, although a number of efforts to bring OA into the spotlight of political attention have been made during the past decade (see “Responses to ocean acidification” section). In studies emanating from the natural sciences, practical examples on initiatives at both international and subnational levels to address OA problems can indeed be found (cf. Cooley et al. 2015; Osborne et al. 2017), but these do not, naturally, engage with the possible contributions of social science research on these matters. The efforts to increase awareness and scientific knowledge notwithstanding, researches on possible political and policy responses in the current social science literature are few and far between. For example, Armstrong et al. (2012) claim that OA has generated very few economic or social science studies in any country, despite the fact that the few studies that *have* been conducted anticipate significant negative impacts on fisheries (Cooley and Doney 2009; Narita et al. 2012), coral habitats (Brander et al. 2012), and marine ecosystem services (Turley et al. 2010). An important and ambititious aim with this review is thus to highlight and help recognize OA as a key issue among disciplines conducting social environmental studies and also to outline potential avenues for future research in the social sciences. By highlighting the major knowledge gaps in the current literature, and suggesting avenues for moving forward, this article thereby hopes to encourage social science research to further engage with this highly relevant and topical problem.

In the following sections of this paper, we review the literature on societal causes to OA (“Causes of ocean acidification” section); the legal, economic and political contexts, and possible mitigation as well as adaptation responses and challenges (“Responses to ocean acidification” section), and we provide concluding remarks including a number of research gaps (“Concluding remarks and research needs” section).

## Causes of ocean acidification

Figure 1 suggests that the human behaviours of multiple types of actors who undertake multiple kinds of activities impact the environment via anthropogenic stressors that influence OA. Many types of human behaviours are currently contributing to the undesirably increasing levels of OA via CO_2_ emissions, from local to global levels, and in sectors as diverse as the production of transport, energy, heating, and food. Figure 1 illustrates the main sectors of activity that generate CO_2_: land-use change; deforestation (increases albedo, but releases CO_2_, and reduces CO_2_ capture creating changes in the “land sink”); and burning fossil fuel and other industries. The extents to which different activities contribute to acidification, however, vary rather substantially between different geographical locales. For example, in a Swedish context, burning fossil fuel and other industries are likely to have a much larger impact than land-use change, while deforestation is probably not relevant at least on average. In the Baltic Sea, emissions from shipping play an increasing role. On the US east-coast, as well as in southern Australia, pollutants and soil erosion are suggested as significant contributors to create acidification hot spots (Kelly et al. 2011). In addition, some other actors may also affect the impacts of OA via interacting stressors (e.g. eutrophication, wastewater discharge, fishing, and varying degrees of water salinity). Finally, some other actors also contribute at multiple scales to make marine ecosystems more vulnerable to the impacts of OA. This is a broader context of issues that we do not discuss here.

Formal and informal institutions form social restrictions to human actions. For example, the market price of fish may influence how much fish people can afford to buy, sell and produce. In contrast, preferences and tastes not only constitute individual restrictions, but they also influence whether people prefer to buy fish or some other type of food. Figure 1 illustrates how both kinds of restrictions provide incentives that steer human action in a direction that can influence the level of OA and its impacts. Hence, if the formal and informal institutions fail to provide the right incentives, the resulting outcome is likely to be an undesirable level of OA because many of the sources of CO_2_ can increase in uncontrolled ways. The literature (e.g. Hepburn 2006) provides substantial evidence that spurious incentives typically result from failures in the formal institutions either through market failure or governance failure. Successful societal responses to OA therefore require addressing both market failure (“Market failure” section) and governance failure (“Governance failure” section), which we are going to explain here.

### Market failure

Local, national and international markets^2^ provide places of exchange for goods such as meat, fish, dairy products, wood products or public transportation, and thus influence the supply and demand for these goods and hence the way they are produced (i.e. use of energy, land-use, industrial activities), which also impacts emissions of CO_2_. For example, global markets for fossil fuels and air transportation affect CO_2_ emissions directly and substantially. Many goods and services traded in markets (e.g. cement) also generate CO_2_ as a by-product and thus contribute indirectly to the increasing levels of OA. While the economic literature on market failures in general is extensive, the results devoted specifically to OA are rare in this discipline, and a search for key word “ocean acidification” in the database ECONLIT (21 March 2018) returned only 18 publications, most of them focusing on impacts rather than causes, with a few exceptions (Fauville et al. 2011; Harrould-Kolieb and Herr 2011; Galaz et al. 2012; Miller et al. 2013).

In theory, markets with perfect competition^3^ could deliver—and allocate in time and space—the amount of OA *optimally* for the long-term global well-being of human society. The level of OA would be *optimal* in the sense that any other level or allocation of OA could only improve the situation for anyone if someone else was made worse off (Arrow 1951; Arrow and Debreu 1954). Hence, economic theory would suggest that levels of OA greater than would be *optimal* for society are essentially due to multiple kinds of market failures^4^ when perfect competition cannot be achieved. Four kinds of market failure seem particularly pervasive in the case of OA. We describe them individually although they typically occur simultaneously.^5^

**Fig. 2:**
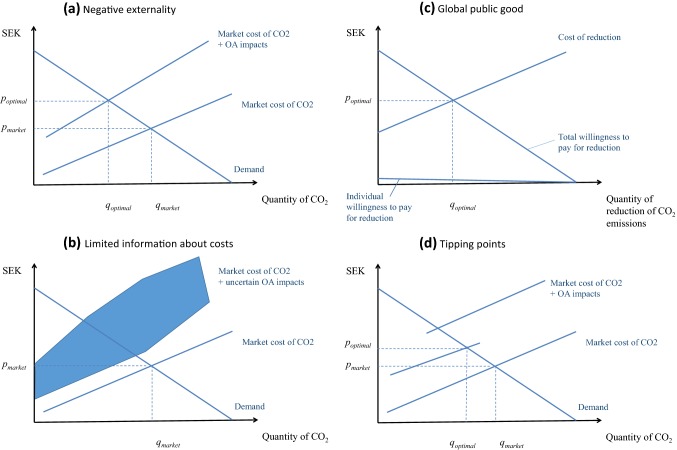
Four market failures causing excessive OA: **a** the market does not account for all the cost associated with emitting CO_2_; **b** there is substantial lack of information about the impacts of OA, so market cost of CO_2_ + OA impacts are represented by an area of potential location of that curve; **c** reducing CO_2_ emissions is a public good so individuals will not be willing to pay so much even though collectively they would benefit, and hence the market will not reduce emissions sufficiently if at all; **d** “tipping points” may occur, such that multiple equilibria are possible for large levels of demand and the system may reach a suboptimal equilibrium. The blue curves represent market demand (marginal benefits, here downward sloping) and market supply (marginal costs, upward sloping), with or without cost of OA impacts. *p*_market_ and *q*_market_ denote, respectively, the price and quantity that will occur spontaneously on the market, while *p*_optimal_ and *q*_optimal_ denote, respectively, the price and quantity that would be optimal

*Negative externality* (Fig. 2a). Property rights to the seas are often ill defined (especially outside exclusive economic zones), and emissions of CO_2_ have impacts far away from their sources. Hence, current CO_2_-emitting activities do not account for the true cost that these emissions generate to people not involved in the market transaction. National policies could provide incentives to internalize the cost of carbon. However, in order to account for the true costs of carbon on global warming, these must also be globally coordinated, address all sources of carbon, and account for climate change including warming *and* other changes due to CO_2_ including acidification effects related to carbon discussed here (Turley and Gattuso 2012; de Campos Rodrigues 2016), but also other effects like hypoxia. Sweden has already in place a substantial set of carbon taxes. Meanwhile these do not cover all emitting industries, they are not globally coordinated and do not account for OA (Sterner and Coria 2012).

*Limited information on costs* (Fig. 2b). Knowledge about the impacts of OA, and—in part—the processes that influence it, is limited and consequently OA-relevant decision-making must deal with substantial uncertainty (Polasky et al. 2011). Armstrong et al. (2012) identified limitations of knowledge that complicate the economic evaluation of the impacts of OA while Brander et al. (2014) refer to knowledge gaps (see Table 1). However, recent development (e.g. Seung et al. 2015; Colt and Knapp 2016; Narita and Rehdanz 2017) indicates that progress is rapidly being made in that area.

**Table 1:** Areas of knowledge limitations and gaps with regard to the impacts of ocean acidification on society (Armstrong et al. 2012; Brander et al. 2014)

Armstrong et al. (2012)	Brander et al. (2014)
(i) effects of OA on ocean ecosystems (structures and functions) (ii) how OA affects/will affect ecosystem services from the sea (iii) economic values of those ecosystem services (iv) methodological limitations with regard to economic valuations of such services (v) only scant knowledge of human preferences for services from the ocean	(i) understanding the relation between changes in the marine environment and socioeconomic impacts (ii) the ecosystem services that have been assessed (iii) the distribution of impacts (iv) the vulnerability of different human populations

*Global public good* (Fig. 2c). The capacity of the oceans to buffer the effects of OA, and the ability of marine ecosystems to adapt and function under changed pH, can both be seen as global public goods, in the sense that any effort by an individual to attain such buffering automatically benefits other individuals equally. Decentralized decision-making in markets generally lead to under-provision of public goods, since the parties generating the public good do not account for the positive effects imposed on others. This dimension of the problem is particularly complex for OA because causes and impacts span the entire planet, implying that no single country can unilaterally address it. Hence regulating the limitation of OA as a public good cannot be achieved unless countries cooperate to implement the agreed upon policy. Such regulation would require lump-sum financial transfers between individuals, which are practically difficult within a nation state, and even more doubtful in an international context (Sandmo 2003); this could, for example, imply that one country would have to compensate other countries that decrease their emissions.

*Tipping points* (Fig. 2d). Ecosystem responses to OA may be rapid, and at the same time influence evolutionary processes (Sunday et al. 2011). Hence, OA will involve both slow and fast processes, which may trigger rapid, substantial and persistent change—so-called *regime shifts*—in marine ecosystems beyond some particular threshold level of OA also called tipping point (Scheffer et al. 2001; Casini et al. 2009; Hughes et al. 2013). Due to unique local environments, heterogeneity, biological adaptation, etc. these tipping points will typically differ in each single location. Several international initiatives aim to identify these changes (www.regimeshifts.org; https://www.resalliance.org/thresholds-db). This implies that some management choices could be irreversible and that the effects of current levels of OA could impact current and future generations very differently. For example, in Sweden, Finland or the Baltic States, OA could interfere with tipping points in the Baltic Sea that trigger eutrophication (Gustafsson et al. 2012) and changes in marine food chains (Tomczak et al. 2012).

### Governance failure

OA interacts with many drivers and outcomes, including climate change, marine biodiversity, and food security, therefore its ecological and social repercussions are embedded in a highly diffuse and complex institutional setting. Several actors and international institutions act in this problem domain, where poor political, administrative, and other institutional arrangements, results in slow progress (Galaz et al. 2012).

Previous research in the environmental social sciences has demonstrated that both the type of political institutions as well as the quality of these play an important role in countries’ environmental performance (Jagers 2007; Min 2015; Povitkina 2018). For example, democratic institutions tend to provide more public good than non-democratic alternatives (e.g. Lake and Baum 2001; Bueno de Mesquita 2003; Acemoglu and Robinson 2006), thus democratic institutions may have better conditions to cope with OA compared to non-democratic countries. However, even democratic institutions sometimes fail to provide public goods. Politicians focusing on re-election do not always benefit the public interest (Besley and Coate 1998; Sterner et al. 2006), as rational, vote-maximizing politicians are unlikely to introduce policies that they fear the electorate could dislike (Page and Shapiro 1983; Stimson et al. 1995; Burstein 2003). Elected leaders often work with short time horizons (Haggard 1991; Sterner et al. 2006; Keefer 2007), while mitigation of, and adaptation to, OA is a truly long-term undertaking. In addition, government policy interventions may result from multiple goals and prioritizations, sometimes contradictory. For example, policies focusing on energy provision or decentralization may even worsen OA if they promote cheap energy or subsidize transport activities that generate more acidification (Anthoff and Hahn 2010; Helm 2010). In addition, for both democratic and non-democratic states, the quality of government (e.g. degree of transparency, impartiality, and corruption) conditions the overall environmental performance (Lægreid and Povitkina 2018). A range of studies have demonstrated the necessity of an uncorrupted state for delivering environmental public goods (Weidner and Jänicke 2002), a capable public administration in implementing environmental policies (Duit 2016), as well as the importance of rule of law (Fredriksson and Mani 2002) and the low level of corruption in securing environmental policy implementation (Pellegrini 2011; Sundström 2015).

While the policies surrounding OA are “characterized by the absence of multilateral agreements for policy coordination among states” (Dimitrov et al. 2007, p. 231) and the Paris Agreement (2015) barely has improved this situation, a few state actors attempt to respond and prepare for the repercussions of OA. For example, states around the Coral Triangle (in the tropical waters of Indonesia, Malaysia, Papua New Guinea, Philippines, and others) collaborate in the region to improve food security and protect marine ecosystems at risk (Fidelman et al. 2012; Rosen and Olsson 2012), in 2015 the G7 countries issued a joint statement pointing towards the risks with OA, and the EU has funded several initiatives to better understand the causes and consequences of OA (cf. Osborne et al. 2017). In addition, several international initiatives (led by non-governmental organizations and the United Nations agencies) exist to create awareness around the problem, synthesize and disseminate scientific information, and try to influence high-level negotiation arenas such as the United Nations Framework Convention on Climate Change (UNFCCC) and the International Atomic Energy Agency (IAEA) (e.g. the Ocean Acidification International Coordination Centre: OA-ICC). Substantial outcomes from these processes, in terms of governmental action to implement concrete policies and policy tools with the capacity to generate behavioural change necessary to prevent OA in specific, are still largely unseen (Galaz et al. 2012; Osborne et al. 2017).

## Responses to ocean acidification

With a few prominent exceptions (e.g. Pacala and Socolow 2004; Cooley and Doney 2009; Harrould-Kolieb and Herr 2011; Kelly et al. 2011; Rau et al. 2012; Billé et al. 2013; Kelly and Caldwell 2013; Colt and Knapp 2016; Narita and Rehdanz 2017), the majority of research on societal responses relevant to OA focuses explicitly on other factors that drive and/or threaten marine systems, notably the overarching concept of climate change. Nonetheless, research on environmental politics and policy (in particular with regards to global climate change) is also highly relevant to address OA, either because we can translate lessons from other empirical areas to fit the problem (e.g. administrative structures, multilateral cooperation, use of policy instruments, etc.), or because addressing one well-researched major problem (CO_2_-induced climate change) also directly affects OA. Table 2 provides an overview of possible responses to OA found in the social science literature.

**Table 2:** Broad categories of suggested responses to OA in the social science literature (e.g. Cooley and Doney 2009; Billé et al. 2013)

Category of response	Target
Mitigation of the main source of OA	Anthropogenic CO_2_ emissions
Mitigation of other greenhouse gas (GHG) emissions	NO_*x*_ and SO_*x*_ emissions
Mitigation of other local/regional factors that contribute to (or compound the effect of) OA	Pollution, eutrophication (phosphor, nitrogen), biodiversity loss
Adaptation of ecosystems and human activities that build resilience to OA	Reducing environmental stress through adjusted fishing quotas and fishery management plans (encouraging multispecies fishing), enhanced electrochemical weathering, increasing aquaculture, labour market adjustments (supporting job transitions from fishery/tourism), social support to local marine-resource-dependent communities in transition
Restoration activity	Developing marine protected areas, enabling the survival of non-commercial species and the preservation of fundamental ecosystem functions and services, reintroduction of coral and marine plants, e.g. seagrasses

We present the Swedish legal framework in place (“The legal framework” section) before we address more specifically mitigation (“Mitigation” section) and adaptation efforts (“Adaptation” section).

### The legal framework

The most prominent governmental instrument used for achieving behavioural change is law; at the international level predominantly as a means to agree on and coordinate action by multiple (state) actors, at the national (and partly EU) level, as a more direct instrument for inducing or forcing behavioural change among individuals and corporations. In the field of environmental protection, legal measures provide the basis both for what is often referred to as ‘command and control’ measures (e.g. technology requirements, emission limits, etc.) and for market-based instruments (e.g. cap and trade schemes).

Multiple legal policy tools targeting CO_2_ emissions are already in place at the regional, national and subnational levels. They are not designed to address OA specifically, but can be marginally adapted to do so (Billé et al. 2013). The main source of OA—CO_2_ emissions—is subject to international agreements, notably the Paris Agreement committing states to make recurring and increasingly ambitious undertakings and implement them in the form of affective measures. Various measures aiming to affect the behaviour of business and industry in relation to CO_2_ emissions are prescribed by EU law. In a similar way, the Convention on Long-range Transboundary Air Pollution (CLRTAP) and several EU legal acts regulate emissions of other important stressors that compound the effects of OA (emissions of SO_*x*_ and NO_*x*_, and eutrophication caused by release of nitrates and phosphates into water). Further, several legal instruments have been developed with the primary, or secondary, aim to protect the marine environment. See Table 3 for overviews. All major EU measures in these areas take the form of directives rather than directly applicable regulations, so member states must transpose them into binding measures at the national level (e.g. Langlet and Mahmoudi 2016; Lindegarth et al. 2016). This transposition generally results in significant variations between member states as to how the EU measures are applied and, in some cases, with respect to how the EU requirements are construed or interpreted.

**Table 3:** EU directives relevant to OA

Directive numbers	Area of regulation covered
2003/87/EC	Emission trading scheme
2009/28/EC	Measures for the promotion of energy from renewable sources
2012/27/EU	energy efficiency
2008/50/EC	Ambient Air Quality
2008/50/EC	National Emission Ceilings
2016/2284/EU	Reduction of national emissions of certain atmospheric pollutants
2000/60/EC	Water Framework
2008/56/EC	Marine Strategy Framework
91/676/EEC	Nitrates (targets water pollution related to nitrates from agricultural sources)
91/271/EEC	Urban Waste Water

Taking Sweden as an example, relevant EU directives are implemented inter alia through the Act (2004:1199) on emission trading, the Air Quality Ordinance (SFS 2010:477), and the Environmental Code (SFS 1998, p. 808). The Environmental code and associated legislation also include various rules for the agriculture sector, targeting eutrophication. The national and local levels have, at least formally speaking, significant possibilities to regulate nutrient leakage through measures pertaining to land use. OA was mentioned briefly in preparatory works to legal acts relating to climate-change mitigation and to protection of the marine environment, without directly leading to specific measures and has received very little attention in the legal literature with a national or regional focus.

Fishing is one of the more prominent stressors that may compound the effects of OA. In contrast to OA, legal aspects of the management of fisheries have been more extensively discussed in the legal literature, both as regards international law and that of the EU, but again hardly ever with regard to how it may interact with OA (Churchill and Owen 2009; Christiernsson et al. 2015; Lado 2016). Within the EU, fisheries are mainly regulated under the EU’s common fisheries policy. There is some limited room for individual member states to regulate fishing in the vicinity of their own coast and conducted by their own fishing fleet (CFP Regulation (EU) No. 1380/2013; Christiernsson et al. 2015). Despite their local significance, these local powers cannot provide a system-level impact on the stressors relevant to OA. OA impacts on fishing have not yet affected decisions on fish quotas or other management measures. Marine protected areas may contribute to increase resilience in ecosystems subject to multiple stressors. They are partly regulated at the EU (e.g. through minimum requirements of protection as part of the Natura 2000 network, and through restrictions posed by EU fisheries policy) and partly at national levels. Typically, these decisions are not either influenced by concerns on OA. However, considerations of climate-change and OA impacts are beginning to be incorporated into the marine spatial planning efforts required by the EU law (Directive 2014/89/EU establishing a framework for maritime spatial planning).

### Mitigation

Mitigation strategies aim to treat the problem at its root by reducing its very causes. In the long term, limiting emissions of CO_2_ to the atmosphere, and reducing the amount of CO_2_ in the atmosphere (Billé et al. 2013), are the only mitigation strategies available to comprehensively address OA (Pacala and Socolow 2004; Cooley and Doney 2009). Hence many (though not all, see Herr et al. 2014) of the policies targeting climate change already in place will also mitigate OA. However, although increasing anthropogenic inputs via CO_2_ emissions drive acidification globally, local acidification is also the result of non-atmospheric stressors on the local level such as abiotic processes, eutrophication, hypoxia, upwelling events bringing low-pH water to near-shore areas, sulphur dioxide precipitation, and runoff from acidic fertilizers caused by agriculture, mineral extraction, and poor ecosystem management (cf. Kelly et al. 2011). Therefore, mitigating OA requires concerted efforts on several levels—from the local to the global. On the one hand, *global* mitigation strategies require a wide range of efforts on the global and international scale, several of which have already been initiated through multilateral cooperation such as the 2015 Paris Agreement (UNFCCC 2016), the 1992 Convention on Biological Diversity (CBD 1992), and the 2013 Kiruna Declaration (AC 2013). On the other hand, multilateral processes are typically complex and politically contested, requiring the involvement of a range of different actors and therefore not “quick fixes” (Sterner et al. 2006), partly due to the anarchic nature of the international political system (cf. Bäckstrand 2006). Indeed, as pointed out by Osborne et al. (2017, p. 404) “the required actions to mitigate and adapt to ocean acidification have yet to be incorporated in international policy”. From this follows that short-term mitigation responses to OA (including both CO_2_-emissions and non-CO_2_ stressors)—as well as the social science research on these matters—should rather focus on developing policy at the local and regional levels, where existing management structures and legal systems are more likely to effectively address these challenges (Cooley and Doney 2009; Kelly et al. 2011; Cooley et al. 2015). Also, internationally agreed measures almost invariably need to be translated into (legal) measures at the national or subnational level to have direct impact on the behaviour of individuals and firms, thereby further strengthening the case for paying attention to measures at these levels as well as the EU level where measures can have similar effects.

Although not overwhelming, some examples on such national and subnational efforts to both adapt to, and mitigate future OA problems, do exist in the more recent literature. One prominent example put forward on political responses to OA concerns the collapse of the oyster industry on the US west coast. This, in turn, sparked reactions on both the local level, building networks among researchers and stakeholders to monitor changes and disseminate information, as well as the state level of government, integrating OA into long-term state resource management plans (Osborne et al. 2017). In Maine, on the US east-coast, a similar problem led to similar network-building reactions on the local and state levels, and a number of state bills addressing the problem with nutrient pollution from farms and septic systems have since been put forward (Cooley et al. 2015). In addition, in the case of the US, it has been suggested to use the Federal Clean Water Act to limit OA-inducing pollutants as a national-level response to acidification, although its authority is limited to land-based point source pollutants (cf. Kelly et al. 2011), and the adoption in the US of the Federal Ocean Acidification Research and Monitoring Act of 2009 are examples on national-level responses spurred by the increasing public attention to the OA problem (Osborne et al. 2017). A range of examples on similar efforts to build knowledge, natural science research capacity, and political attention also exists outside the US: the Brazil OA Research Network (BROA), the Biological Impacts of OA Program (BIOACID) in Germany, and the United Kingdom’s Ocean Acidification Research Programme (UKOA) to name but a few (Cooley et al. 2015). In parallel, also cross-national networks, such as the European Project on Ocean Acidification (EPOCA), and the MedSeA Project to study OA in the Mediterranean Sea funded by the European Commission have been launched during the past 10 years.

However, although these efforts have served to raise awareness and knowledge about the problem among policy makers, a next step in mitigating OA is the real-life design and implementation of concrete policy tools, capable of addressing practices and behaviours that contribute to the anthropogenic stressors for OA. This, however, presents policy makers with significant challenges as behavioural change is not easily governed. In these endeavours, social science research can and will play an important role, for example, in the designing of effective policy tools, simultaneously utilizing opportunities and negotiating barriers for successful policy development and implementation.

We discuss here the challenges of changing behaviour with policy tools and the rationale behind different types of policy tools.

#### Changing behaviour with policy tools

OA exhibits the characteristics of a classic collective-action problem: lack of cooperation among actors responsible for OA results in suboptimal outcomes for the collective (Olson 1965). As Fig. 2c illustrates, limiting CO_2_, and thus OA, will rarely be provided in sufficient quantity, (if at all), because of the difficulties of aggregating all actors’ willingness to pay for it. There is an imminent risk of free-riding arising regardless of whether or not the collective willingness-to-pay for an additional limitation of CO_2_ emissions is large (e.g. Ostrom 1990). Significant voluntary behavioural changes are therefore unlikely among individuals. The same holds true regarding potential appropriate responses to the negative externalities, large uncertainties, and the risk of tipping points involved in OA (Fig. 2a, b, d). Some form of intentional, third party coordination or *governmental coercion* (Mansbridge 2014) is usually necessary to address market failures and initiate cooperation, especially when the number of involved actors is very large, dispersed over vast geographical areas, and therefore mostly anonymous to each other. Such governmental coercion usually takes the form of various policy tools aimed at changing the incentive structures governing behavioural choices, either by increasing the attractiveness of preferred behaviour or by exacerbating the negative impact of an undesired behavioural choice.

The global nature of OA creates a need for global cooperation but the lack of a supranational authority and limited enforcement mechanisms at that scale suggest that legal measures to initiate multilateral cooperation must take the form of voluntary entered into agreements and treaties. Thus, this set-up is largely dependent on the will and the ability of individual states. This can be extremely tricky to achieve (see e.g. the recent withdrawal of the US from the Paris Agreement), in particular agreements of how costs and benefits should be distributed across national borders, and negotiating multiple free-riding problems (cf. Barrett 2003). Although not directed towards OA specifically, several international legal regimes addressing the direct and indirect sources of OA are already in place. Global strategies for CO_2_ mitigation arise from the UNFCCC and subsequent instruments, including the Kyoto Protocol and the recent Paris Agreement. Despite increasing knowledge about OA, the global climate regime contains no provisions explicitly aimed at or related to OA. This has triggered proposals for the elaboration of a specific international agreement focusing on combating OA (Kim 2012) or for at least highlighting OA as a problem separate from climate change within the present agreements (Herr et al. 2014). These global-level agreements must be implemented in domestic (or in the case of the EU, regional) legal systems to directly affect the legal situation of individuals or companies. Implementation leaves significant discretion for individual states to choose instruments and methods that are consistent with their legal traditions and political preferences resulting in diverse rules and mechanisms subsequently employed in different jurisdictions.

Governments across the world, have proposed, developed, and implemented many pro-environmental policy measures in their attempts to overcome large-scale collective-action problems and, thus, to induce positive individual-level behavioural changes (Jordan 2005; Sterner and Coria 2012; IPCC 2014). Rather than focusing on OA per se, relevant current literature is concerned with policy measures aimed at lowering CO_2_ emissions for the purpose of mitigating climate change.

#### Regulatory, market-based and informative policy tools^6^

In general, economic policy responses aim to alter incentive structures by directly addressing market failures (i.e. negative externalities, information deficits, public-goods provisions, etc.) that give rise to unwanted behavioural patterns. Typically, regulating CO_2_ emissions, or emissions of other GHG’s and pollutants, using economic policy instruments is done through quantity regulation (the amount that should be produced), price regulation (taxes), or mixed regulation (e.g. Cap and Trade; see Hepburn 2006). Such policies have been extensively studied in the context of climate change reduction, but, in principle, OA mitigation could be achieved by the same means. The use of CO_2_ taxes is widely regarded as one of the most cost-effective means of limiting emissions and changing behaviour, and has been implemented in Sweden since 1991 with gradually increasing public support (Jagers and Hammar 2009; Jagers and Matti 2010). Similarly, taxes on the commercial use of fertilizers and pesticides as well as on (land-based) NO_*x*_ and SO_*x*_ emissions have been in place since the early 1990s.

Other types of economic policy tools that currently are, or could be, directed towards reductions of emission include pull-instruments that subsidize more favourable alternatives. These can target the production of alternative energy sources (e.g. feed-in-tariffs for biofuels or other renewables) and consumer behaviour (e.g. eco-car subsidies) (Söderberg 2011). Several such subsidies are already implemented in Sweden.

In contrast to regulations and market-based instruments, informative policy tools serve to highlight the problem aiming to initiate voluntary action and increase support for implementing more coercive policy tools. Informative policy tools can also decrease information asymmetries between different actors. Examples of information instruments include eco-labelling or certification schemes for products or technologies, and collection and disclosure of data on identified greenhouse gas emissions by significant polluters (Krarup and Russell 2005). Such types of policy can also trigger changes in social norms if the information can change the perception of large groups in society about what is accepted behaviour and what is not (Nyborg et al. 2016). This is, however, complex as consumers have many different reasons for choosing an eco-labelled product, which are not always well correlated with information/knowledge about environmental impacts (Jagers et al. 2016; Jonell et al. 2016). Nonetheless, as research demonstrates that people are largely unaware of OA and its potential consequences (Leiserowitz et al. 2010; Frisch et al. 2015; Capstick et al. 2016), and people in general are unsupportive of solutions to unfamiliar problems (e.g. Stern et al. 1995), better information on OA is a potentially important tool to legitimizing mitigation policies.

A second aspect of information relates to appropriate provision of research for understanding the mechanisms underlying OA and its impacts. Identifying what levels of OA might be acceptable would require assessing all the trade-offs between valuable economic activities that generate OA (i.e. CO_2_), the harm to society caused by the resulting increase in OA but also the economic values and losses arising from activities that amplify the negative effects of OA. Consequently, the optimal price of CO_2_ to society cannot be calculated, making it difficult to calibrate any policy instrument targeting OA in Sweden. This task is currently intractable given the relatively low levels of knowledge in particular about the impacts of OA. More knowledge is needed about the problem and its causes and about ways of targeting the problem (Armstrong et al. 2012; Turley and Gattuso 2012; Brander et al. 2014). As with OA mitigation, however, research is a public good, which necessitates governmental intervention.

### Adaptation

Adaptation strategies focus on treating the *symptoms* of OA by adjusting natural or human systems such that damage is reduced, and/or beneficial opportunities taken advantage of (Adger et al. 2007). Adaptation does not target the causes of the problem but rather aims to maintain social well-being in spite of OA. These types of strategy are likely to be easier to apply than mitigation strategies, especially in the short term, because symptoms of OA are problematic at local and regional levels where people can also address them (Cooley and Doney 2009). Hence adaptation usually requires less coordination effort than global mitigation policies, and those efforts are typically located at the local and regional level where relevant national institutions are usually already in place. Nonetheless, practical examples of OA adaptation remain scarce, and the barriers to negotiate are in many ways the same as those for mitigation strategies, including collective-action problems and information deficits. Three broad types of adaptation strategy can be identified, spanning structural–physical, social, and institutional adaptation. All of these require further government policies and programmes to be initiated and funded (IPCC 2014). In addition to the main strategies outlined in this section, broader adaptation potential can be increased through capacity-building activities such as infrastructural improvements, increasing institutional capacity, information, and access to resources (Smit et al. 2001).

#### Strategies to reduce the negative impacts of acidification on marine ecosystems

Ecosystem resilience to OA can be strengthened in the short term by alleviating pressure from other stressors, e.g. by reducing the rate and magnitude of eutrophication, pollution, and fishing. Ecosystems with higher diversity are more resilient to other forms of environmental stress, including OA as available data suggests. Hence, ecological restoration may be a particularly valuable tool in maintaining/increasing diversity and thereby increasing resilience to OA (Worm et al. 2006), implying synergies with efforts to conserve or restore biodiversity (e.g. CBD 1992). As long as ecosystems remain within critical thresholds, human activities such as fisheries and aquaculture can adapt to change. For example, the effects of OA and warming on shallow seagrass ecosystems are similar to the effects of eutrophication and increased fishing pressure (Alsterberg et al. 2013); thus, reducing eutrophication and fishing pressure may (in the short term) directly offset the effects of OA and warming. Furthermore, counteracting OA by alkalinization may be useful in hotspots such as coastal environments but seems to have very limited potential and feasibility at larger scales (e.g. Billé et al. 2013; Weatherdon et al. 2015). In two recent publications, Osborne et al. (2017) as well as Cooley et al. (2015) outline further adaptation strategies.

#### Strategies to adapt the way society organizes

The impacts of OA are likely to affect ecosystem services produced in the oceans. Turley and Gattuso (2012) list broad categories of OA impacts on these services including fisheries, aquaculture and food security, coastal protection, tourism, climate regulation, and carbon storage. OA may strengthen some services and reduce others. Negatively affected provisioning services (e.g. landings of fish for human consumption) will likely lead to price increases and impact on food security. A key question is then to what degree those fish may be substituted? For example, fish can become relatively more expensive than other sources of protein and market forces will then automatically steer the economy towards less dependence on fish. This may, however, increase production of substitutes like land animals that can generate higher release of CO_2_ compared to fish, thus further adding to climate change and OA.

If such transition processes are slow and costly, progress may be accelerated by: (i) compensating losers (e.g. fishermen), (ii) providing transitional support, and/or (iii) stimulating innovation to accelerate emergence of alternatives and technical replacement solutions. However, substitutes for the goods or services damaged by OA may not always be available (e.g. loss of fisheries and tourism income due to degradation of high diversity coral reef systems; Worm et al. 2006). Hence, societal priorities will be forced to target less-damaged (or undamaged) goods and services. In the extreme case when life-support systems are affected and no substitute is available, this could have catastrophic impacts on human well-being (although such impacts are perhaps unlikely to arise from OA). This is true even if the absence of substitutes is temporary. These types of response can be deployed either by individual countries or through multilateral cooperation.

#### Strategies to compensate people who lose from ocean acidification

Changes in the provision of marine ecosystem services arising from OA will likely generate redistributions of resources between user groups. For example, the different responses of two Arctic fishing communities to the disappearance of north-west Atlantic cod stocks led to two very different outcomes—one community lost substantially while the other was able to target other species and increased income (McCain et al. 2016). The opportunity for such responses may, however, be limited if OA has negative impacts on most fish species. Viable adaptation strategies include compensation to disadvantaged groups, and/or helping them adapt to the new situation by stimulating education, investment, etc. However, avoiding the establishment of spurious incentives that effectively reward some sections of society for not managing the change is vital, and therefore such strategies should be transitional (Dixit and Londregan 1995).

## Concluding remarks and research needs

This review outlined the societal aspects of OA aiming to identify its major social and political causes, and subsequently also the primary mitigation and adaptation responses needed to reduce future OA and alleviate current and future consequences if not being mitigated properly. In regards to (Q1): *What are the primary causes of anthropogenic ocean acidification from a social science perspective?* We found that the primary causes of anthropogenic OA from a social science perspective relate to governance and market failures. These failures are exacerbated by the global nature of OA which requires cooperation among states to address it. However, the prospects for coordinated policy efforts, and the extent to which policy diffusion or transfer is possible, are unclear due to contextual variations. In addition, substantial impacts of OA on the generation of ecosystem services and hence on human well-being will likely alter resource distribution between individuals, which may seem unfair for those who lose out and especially among those who lose out while having contributed least to the occurrence of the problem. Further, assuming linear changes, the rate of adaptation to OA in our human systems would have at least to keep pace with rates of ecological change in order for people to continue to derive benefits from key marine ecosystem services. With the potential for ecological tipping points, the rate of adaptation may even have to be much more rapid to avoid negative societal impacts. Our review highlights the current scant knowledge related to the extents and impacts of different market and governance failures in relation to OA, and how these interact with each other.

This review also outlined multiple ways in which (Q2) *society can respond to ocean acidification*, with a special focus on a Swedish context. Little is currently known about the appropriateness of various existing policies, legal provisions, mechanisms, and administrative systems that address either the main cause of OA (i.e. increasing atmospheric CO_2_), or the additional stressors that may influence resilience and adaptation. This lack of knowledge prevents informed assessment of the current institutional framework within which OA arises, and subsequently the design of additional or modified measures to deal with OA. Our review also clearly demonstrates the lack of a comprehensive overview of mitigation structures, in Sweden and elsewhere.

From a Swedish perspective, reducing locally managed anthropogenic pressures (e.g. fisheries) could increase resilience to OA in the short run. Thus, devising strategies for changing local management practices for non-OA stressors may reduce threats from OA to key ecosystem services. However, this is likely not a long-term solution. The global extent of OA, its complex social–ecological dynamics involving potential tipping points, the clear role of anthropogenic CO_2_ emissions to worsen it, the large uncertainties associated with most of its dimensions, and the potentially very large impacts, all together speak for a precautionary approach to address OA (see e.g. Crépin and Folke 2015). While current knowledge of the problem is alarming enough to justify putting in place substantial mitigation policies, better knowledge about the socioeconomic dimensions involved in OA would contribute to policy improvements.

Finally, we identified *major knowledge gaps and research* needs with regard to the future study of OA (Q3), which we summarize in Table 4.

**Table 4:** Research needs in social sciences with regard to ocean acidification, key domains are highlighted

Research needs	
• Identify the types and magnitude of *market failures* relevant to OA, their consequences, quantify their relative importance and interactions among them • Analyse the coherency and/or conflicts between the *national*, *EU*, *and international legal and administrative systems* relevant for OA • Further explore contradictions, overlaps, and gaps in *existing institutional arrangements* that cause OA, and how they can be amended, focusing on the legal and administrative feasibility including the degree of readiness to pursue required changes among concerned actors • *Build a schematic understanding* of how the existing regulations and management structures link to OA, and to each other and how current policy tools reinforce (or counteract) each other in the aim of changing human behaviour • Analyse whether increased *public knowledge* of the causes and consequences of OA, and policy tools to address this problem, could increase public policy support • Understand the importance of *political*–*economic context* for policy support, and how this interacts with *individual*-*level mechanisms* driving policy support and behavioural change • Develop knowledge on how to foster, evaluate and implement increased *ecosystem resilience to OA through habitat repair and protection* • Improved *resource allocation strategies* (e.g. transitional compensation systems, support to new activities, etc.), and identification of winners and losers among societal groups • Investigate the *impacts of different measures on incentives for innovation* in order to avoid lock-ins and maintenance of old structures that are not adapted to the new situation	

## Footnotes

1 The term “ocean acidification” is used to describe the shift in the acid–alkaline balance of seawater towards more acidic (less alkaline) values. It is caused by increasing levels of CO_2_ in seawater due to uptake of CO_2_ from the atmosphere.

2 A market is an institution, where goods, including services and information, can be exchanged between buyers and sellers. This definition includes traditional market places but also online trade and other institutions framing the exchange of goods and services.

3 A perfect competition market has a large number of perfectly informed rational buyers and sellers, with well-defined property rights, no power to set the price, homogenous products, no barrier to entry or exit, no transaction costs, no impacts on third party, no economies of scale. This combination occurs very rarely in real life.

4 Market failures (Bator 1958) are often linked to (Hoch and Loewenstein 1991), information asymmetries (Stiglitz 1998), non-competitive markets (Tirole 1988), principal–agent problems (Hart and Holmstrom 1987), externalities (Laffont 2008), or public goods (Baumol and Oates 1988).

5 Another aspect not discussed here is that CO_2_ can be characterized as a non-point source pollutant. While such pollutants are often only measurable after they have entered the environment (making polluting sources costly or impossible to identify; Kampas and White 2004), CO_2_ production can be identified and quantified readily at source. However, multitudes of small sources are still burdensome to detect, and individual actors can typically only address a tiny part of the problem, which provides less incentive to take action against them.

6 An alternative would have been, e.g. to use Vedung’s (1998) seminal tripartite classification of policy instruments as sticks, carrots and sermons.
